# Track Fusion Fractional Kalman Filter for the Multisensor Descriptor Fractional Systems

**DOI:** 10.1155/2022/9637801

**Published:** 2022-08-24

**Authors:** Bo Zhang, Haibin Shen, Guangming Yan, Xiaojun Sun

**Affiliations:** Electrical Engineering Institute, Heilongjiang University, Harbin 150080, China

## Abstract

The purpose of this study was to investigate the state estimation problem for the multisensor descriptor fractional systems. Firstly, the descriptor fractional order system was transformed into two nondescriptor fractional order subsystem based on the singular value decomposition method; then, the descriptor fractional Kalman filters for the subsystems were proposed based on projection theory, which effectively solved the state estimation problem of the descriptor fractional order system with singular matrix; on this basis, the track fusion fractional Kalman filter of the multisensor descriptor fractional system is proposed by using the track fusion algorithm. The state estimation accuracy of multisensor descriptor fractional order systems is greatly improved. Simulation results show the effectiveness of the proposed algorithm.

## 1. Introduction

Descriptor system theory is an independent branch of modern control theory, which began to form and gradually developed in the 1970s. It has important application in the mechanical system [1] and circuit system [2]. Due to the complexity and uncertainty of its system, the research on descriptor systems is relatively slow. With the passage of time, the research on normal systems has become more and more perfect, and people begin to pay attention to descriptor systems. In just a few decades, descriptor systems have also made good progress [3, 4]. A fractional descriptor reduced-order nonlinear observers for a class of fractional descriptor continuous-time nonlinear systems was proposed by Kaczorek [3]. Necessary and sufficient conditions for the positivity of descriptor linear and sufficient conditions for nonlinear systems were established by Kaczorek [4]. A new algorithm for generalized second order systems was proposed by using nonpolynomial spline technique [5].

The fractional order Kalman filter combines the conventional integer order Kalman filter with the fractional order algorithm to obtain a Kalman filter suitable for fractional order systems [6–8]. The fractional Kalman filters were proposed by Koh et al. [9–11]. The State estimation problem was solved for the fractional-order systems with coloured measurement noise [12, 13]. Recently, many fractional Kalman filters with unknown information were presented. The unknown prior information, polytopic uncertainties in the finite frequency domain, and the direct and networked measurements were considered by Liu et al. [14–16]. The time-delay in the observation signal and unknown input were studied in refs [17, 18]. For the nonlinear fractional Gaussian system, the fractional particle filters were systematically investigated in refs [19, 20]. At present, the descriptor fractional system has been widely applied to many fields such as electrical circuits [21, 22] and sensor fault estimation [23]. However, the filtering problem is seldom studied for the descriptor fractional system.

Multisensor information fusion filtering theory is an important branch of multisensor information fusion, which is a new frontier subject [24, 25]. It is the cross field of multisensor data fusion and Kalman filtering theory [26]. Track fusion algorithm is a globally suboptimal weighted state fusion algorithm. Compared with other algorithms, although it can not get the optimal solution, it is also used by more and more people because of its simplicity, convenience, and small amount of calculation [27].

In this paper, based on the existing fractional filtering theory, a descriptor system track fusion fractional Kalman filter is proposed, which will effectively solve the problem of state estimation and fusion estimation of descriptor fractional order systems. The simulation results show the effectiveness and feasibility of the proposed algorithm.

The remainder of this paper is arranged as follows: in Section 2, the linear discrete descriptor fractional system is provided, and it is transformed into two normal subsystems. The local and track fusion fractional Kalman filters for the descriptor systems are introduced in Section 3, and the simulation example analysis is presented in Section 4. Finally, the conclusion is drawn in Section 5.

## 2. Problem Formulation

Consider the multisensor linear discrete descriptor fractional systems with *L* sensors.

(1) $$\begin{array}{c} E\Delta^{\gamma }x\left(k+1\right)=A_{\alpha }x\left(k\right)+w\left(k\right), \end{array}$$

(2) $$\begin{array}{c} x\left(k+1\right)=\Delta^{\gamma }x\left(k+1\right)-\sum_{j=1}^{k+1}{\left(-1\right)}^{j}\gamma_{j}x\left(k+1-j\right), \end{array}$$

(3) $$\begin{array}{c} y_{i}\left(k+1\right)=C_{i}x\left(k+1\right)+v_{i}\left(k+1\right),\;i=1,\ldots ,L, \end{array}$$

where *γ* is the fractional order, *x*(*k*) ∈ *R*^*n*^ and *y*_*i*_(*k*) ∈ *R*^*m*^ are the state and the measurement of *i* th sensor, *A*_*α*_ and *C*_*i*_ are constant matrices.

(4) $$\begin{array}{c} \gamma_{k}=\text{diag}\left[\left(\begin{array}{c} p_{1}\\ k \end{array}\right)\ldots \ldots \left(\begin{array}{c} p_{n}\\ k \end{array}\right)\right]\text{ ,}\\ \Delta^{\gamma }x\left(k+1\right)=\left[\begin{array}{c} \Delta^{\gamma }x^{\left(1\right)}\left(k+1\right)\\ \vdots \\ \Delta^{\gamma }x^{\left(n\right)}\left(k+1\right) \end{array}\right], \end{array}$$

where *p*_1_,…*p*_*n*_ denotes the orders of state equation, *n* denotes the dimension of state equation, $\left(\begin{array}{c} p_{l}\\ j \end{array}\right)$ denotes combination $\left(\begin{array}{c} p_{l}\\ j \end{array}\right)=\left\{\begin{array}{cc} 1, & j=0,\\ p_{l}\left(p_{l}-1\right)\ldots \left(p_{l}-j+1\right)/j!, & j>0, \end{array}\right.$*l* = 1,…, *n*.

**Assumption 1 .**

E is a singular matrix, i.e. rank*E* < *n*, det*E*=0.

**Assumption 2 .**

The system is regular, i.e. ∃*z* ∈ *C* (complex domain), we have det(*zE* − *A*_*α*_) ≠ 0.

**Assumption 3 .**

*w*(*k*) and *v*_*i*_(*k*) are uncorrelated white noise with zero mean and variance matrices *Q*_*w*_ and *R*_*i*_, i.e.

(5) $$\begin{array}{c} Ε\left[w\left(j\right)w^{Τ}\left(j\right)\right]=Q_{w},\\ Ε\left[w\left(k\right)w^{Τ}\left(j\right)\right]=0,\\ Ε\left[{\text{v}}_{i}\left(\text{j}\right)v^{Τ}\left(j\right)\right]=R_{i},\\ Ε\left[{\text{v}}_{i}\left(k\right)v^{Τ}\left(j\right)\right]=0,\\ Ε\left[{\text{v}}_{i}\left(k\right)v^{Τ}\left(j\right)\right]=0. \end{array}$$

with E as the mean sign and T as the transpose symbol.

**Assumption 4 .**

The system is completely observable, i.e.,

(6) $$\begin{array}{c} \text{rank}\left[\begin{array}{c} zE-A_{\alpha }\\ C_{i} \end{array}\right]=n,\\ \text{rank}\left[\begin{array}{c} E\\ C_{i} \end{array}\right]=n. \end{array}$$

**Assumption 5 .**

The initial state *x*(*k*) is independent with *w*(*k*) and *v*_*i*_(*k*).

In order to deduce the Kalman estimator for the descriptor fractional systems, we must transform the descriptor systems into normal systems at first.

From Assumption 1 and 2, it follows that the nonsingular matrices *P* and *Q* are yielded.

(7) $$\begin{array}{c} PEQ=\left[\begin{array}{cc} S_{1} & 0\\ S_{2} & 0 \end{array}\right],\\ PA_{\alpha }Q=\left[\begin{array}{cc} T_{1} & 0\\ T_{2} & T_{3} \end{array}\right],\\ C_{i}Q=\left[\begin{array}{cc} C_{i1} & C_{i2} \end{array}\right]. \end{array}$$

where *S*_1_ is a nondissimilar lower triangular matrix, *T*_1_ ∈ *R*^*n*_1_×*m*_1_^ is a quasilower triangular matrix, *T*_3_ ∈ *R*^*n*_2_×*n*_2_^ is a nondissimilar lower triangular matrix, *n*_1_+*n*_2_=*n* and it is defined as follows:

(8) $$\begin{array}{c} x\left(k\right)=Q\left[\begin{array}{c} x_{1}\left(k\right)\\ x_{2}\left(k\right) \end{array}\right],\\ Pw\left(k\right)=\left[\begin{array}{c} w_{1}\left(k\right)\\ w_{2}\left(k\right) \end{array}\right], \end{array}$$

then (1) and (3) are transformed into the following subsystems:

(9) $$\begin{array}{c} \Delta^{\gamma }x_{1}\left(k+1\right)={\overset{¯}{A}}_{\alpha }x_{1}\left(k\right)+S_{1}^{-1}w_{1}\left(k\right). \end{array}$$

(10) $$\begin{array}{c} y_{i1}\left(k\right)={\overset{¯}{C}}_{i}x_{1}\left(k\right)+\eta_{i}\left(k\right), \end{array}$$

(11) $$\begin{array}{c} x_{2}\left(k\right)=T_{3}^{-1}\left(S_{2}S_{1}^{-1}T_{1}-T_{2}\right)x_{1}\left(k\right)+T_{3}^{-1}S_{2}S_{1}^{-1}w_{1}\left(k\right)-T_{3}^{-1}w_{2}\left(k\right). \end{array}$$

where

(12) $$\begin{array}{c} {\overset{¯}{A}}_{\alpha }=S_{1}^{-1}T_{1},\\ {\overset{¯}{C}}_{i}=C_{i1}+C_{i2}T_{3}^{-1}S_{2}S_{1}^{-1}T_{1}-C_{i2}T_{3}^{-1}T_{2},\\ \eta_{i}\left(k\right)=C_{i2}T_{3}^{-1}S_{2}S_{1}^{-1}w_{1}\left(k\right)-C_{i2}T_{3}^{-1}T_{2}w_{2}\left(k\right)+v_{i}\left(k\right). \end{array}$$

From (9), (10) and (12), we known that the input noise and the measurement noise are correlated, i.e.,

(13) $$\begin{array}{c} S_{i}=Ε\left[w_{1}\left(k\right)\eta_{i}^{Τ}\left(k\right)\right]=Q_{1}{\left(C_{i2}T_{3}^{-1}S_{2}S_{1}^{-1}\right)}^{Τ}, \end{array}$$

with *Q*_1_ as the variance of *w*_1_(*k*).

Formally adding a term equal to zero to the right of (9), it follows that

(14) $$\begin{array}{c} \Delta^{\gamma }x_{1}\left(k+1\right)={\overset{¯}{A}}_{\alpha }x_{1}\left(k\right)+S_{1}^{-1}w_{1}\left(k\right)+J_{i}\left[y_{i1}\left(k\right)-{\overset{¯}{C}}_{i}x_{1}\left(k\right)-\eta_{i}\left(k\right)\right]. \end{array}$$

with undetermined *n*_1_ × *m* matrix *J*_*i*_, setting ${\tilde{A}}_{\alpha i}={\overset{¯}{A}}_{\alpha }-J_{i}{\overset{¯}{C}}_{i}$, ${\tilde{w}}_{i1}\left(k\right)=S_{1}^{-1}w_{1}\left(k\right)-J_{i}\eta_{i}\left(k\right)$, we have the following state equation:

(15) $$\begin{array}{c} \Delta^{\gamma }x_{1}\left(k+1\right)={\tilde{A}}_{\alpha i}x_{1}\left(k\right)+J_{i}y_{1}\left(k\right)+{\tilde{w}}_{1}\left(k\right), \end{array}$$

and the measurement equation is also (10). Noting $Ε{\tilde{w}}_{1}\left(k\right)=0$ that, and

(16) $$\begin{array}{c} Ε\left[{\tilde{w}}_{1}\left(k\right)\eta_{i}^{T}\left(j\right)\right]=\left[S_{1}^{-1}Q_{w1}{\left(C_{i2}T_{3}^{-1}S_{2}S_{1}^{-1}\right)}^{Τ}-J_{i}Q_{\eta i}\right]\delta_{kj}. \end{array}$$

Making *J*_*i*_ as

(17) $$\begin{array}{c} J_{i}=S_{1}^{-1}Q_{w1}{\left(C_{i2}T_{3}^{-1}S_{2}S_{1}^{-1}\right)}^{T}Q_{\eta i}^{-1}. \end{array}$$

we have $E\left[{\tilde{w}}_{1}\left(k\right)\eta_{i}^{T}\left(j\right)\right]=0$, i.e. ${\tilde{w}}_{1}\left(k\right)$ and *η*_*i*_(*t*) are uncorrelated, it is easily known that the covariance matrix of ${\tilde{w}}_{1}\left(k\right)$ is

(18) $$\begin{array}{c} E\left[{\tilde{w}}_{1}\left(k\right){\tilde{w}}^{T}\left(j\right)\right]=\left[S_{1}^{-1}Q_{w1}{\left(S_{1}^{-1}\right)}^{T}-S_{1}^{-1}Q_{w1}{\left(C_{i2}T_{3}^{-1}S_{2}S_{1}^{-1}\right)}^{T}Q_{\eta i}^{-1}C_{i2}T_{3}^{-1}S_{2}S_{1}^{-1}{\left(S_{1}^{-1}Q_{w1}\right)}^{T}\right]\delta_{kj}. \end{array}$$

so ${\tilde{w}}_{1}\left(k\right)$ is a white noise with zero mean and variance matrix *S*_1_^−1^*Q*_*w*1_(*S*_1_^−1^)^*T*^ − *S*_1_^−1^*Q*_*w*1_(*C*_*i*2_*T*_3_^−1^*S*_2_*S*_1_^−1^)^*T*^*Q*_*ηi*_^−1^*C*_*i*2_*T*_3_^−1^*S*_2_*S*_1_^−1^(*S*_1_^−1^*Q*_*w*1_)^*T*^ and is uncorrelated with white noise *η*_*i*_(*k*).

The descriptor fractional Kalman filtering problem is to find the local and track fusion linear minimum variance estimator ${\hat{x}}_{i}\left(k|k\right)=\left[\begin{array}{cc} {\hat{x}}_{i1}^{T}\left(k|k\right) & {\hat{x}}_{i2}^{T}\left(k|k\right) \end{array}\right]$ and ${\hat{x}}_{0}\left(k|k\right)=\left[\begin{array}{cc} {\hat{x}}_{01}^{T}\left(k|k\right) & {\hat{x}}_{02}^{T}\left(k|k\right) \end{array}\right]$ for the state *x*(*k*) based on the measurements *y*_*i*_(1),…, *y*_*i*_(*k*).

## 3. Track Fusion Fractional Kalman Filter for the Descriptor System

**Lemma 1 (sec [11]).**

$\left({\tilde{A}}_{\alpha i},{\overset{¯}{C}}_{i}\right)$ is a completely observable yes.

**Proof**

See Ref. [11].

**Theorem 1 .**

For the fractional subsystem 1 (2), (10), and (15) with white uncorrelated noises, we have the local recursive fractional Kalman filter.

(19) $$\begin{array}{c} {\hat{x}}_{i1}\left(k|k\right)={\hat{x}}_{i1}\left(k|k-1\right)+K_{i1}\left(k\right)\left[y_{i1}\left(k\right)-{\overset{¯}{C}}_{i}{\hat{x}}_{i1}\left(k|k-1\right)\right], \end{array}$$

(20) $$\begin{array}{c} \Delta^{\gamma }{\hat{x}}_{i1}\left(k|k-1\right)={\tilde{A}}_{\alpha i}{\hat{x}}_{i1}\left(k-1|k-1\right)+J_{i}y_{i1}\left(k-1\right) \end{array}$$

(21) $$\begin{array}{c} {\hat{x}}_{i1}\left(k|k-1\right)=\Delta^{\gamma }{\hat{x}}_{i1}\left(k|k-1\right)-\sum_{j=1}^{k}{\left(-1\right)}^{j}\gamma_{j}{\hat{x}}_{i1}\left(k-j|k-j\right), \end{array}$$

(22) $$\begin{array}{c} P_{i1}\left(k|k-1\right)=\left({\tilde{A}}_{\alpha i}+\gamma_{1}\right)p_{i1}\left(k-1\;|\;k-1\right){\left({\tilde{A}}_{\alpha i}+\gamma_{1}\right)}^{T}+\sum_{j=2}^{k}\gamma_{j}P_{i1}\left(k-1|k-1\right)\gamma_{j}^{Τ}, \end{array}$$

(23) $$\begin{array}{c} P_{i1}\left(k|k\right)=\left(I-K_{i1}\left(k\right){\overset{¯}{C}}_{i}\right)P_{i1}\left(k|k-1\right), \end{array}$$

(24) $$\begin{array}{c} K_{i1}\left(k\right)=P_{i1}\left(k|k-1\right){\overset{¯}{C}}_{i}^{T}{\left[{\overset{¯}{C}}_{i}P_{i1}\left(k|k-1\right){\overset{¯}{C}}_{i}^{T}+Q_{\eta i}\right]}^{-1}. \end{array}$$

with the initial value ${\hat{x}}_{i1}\left(0|0\right)={\hat{x}}_{01}$, *P*_*i*1_(0*|*0)=*P*_01_.

**Proof**

Applying the projection theorem yields to the linear minimum mean square error estimation [11],

(25) $$\begin{array}{c} {\hat{x}}_{i1}\left(k|k-1\right)=\hat{Ε}\left[x_{1}\left(k\right)|Y_{i}^{\left(k-1\right)}\right]=\hat{Ε}\left[\begin{array}{c} {\tilde{A}}_{\alpha i}x_{1}\left(k-1\right)+J_{i}y_{i1}\left(k-1\right)+{\tilde{w}}_{i1}\left(k-1\right)-\\ \sum_{j=1}^{k}{\left(-1\right)}^{j}\gamma_{j}\left[x_{1}\left(k-j\right)|Y_{i}^{k-1}\right] \end{array}\right]={\tilde{A}}_{\alpha i}\hat{Ε}\left[x_{1}\left(k-1\right)|Y_{i}^{k-1}\right]+J_{i}\hat{Ε}\left[y_{i1}\left(k-1\right)|Y_{i}^{k-1}\right]-\sum_{j=1}^{k}{\left(-1\right)}^{j}\gamma_{j}\hat{Ε}\left[x_{1}\left(k-j\right)|Y_{i}^{\left(k-1\right)}\right]. \end{array}$$

where *Y*_*i*_^(*k*)^ denotes the state space constructed by *y*_*i*1_(1),…, *y*_*i*1_(*k*), and $\hat{Ε}\left[x_{1}\left(k\right)Y_{i}^{\left(k-1\right)}\right]$ denotes the projection of *x*_1_(*k*) based on *Y*_*i*_^(*k* − 1^.

From the linear properties of conditional expectation, we have the following equation [11]:

(26) $$\begin{array}{c} {\hat{x}}_{i1}\left(k|k-1\right)={\tilde{A}}_{\alpha i}{\hat{x}}_{i1}\left(k-1|k-1\right)+Jy_{i1}\left(k-1\right)-\sum_{j=1}^{k}{\left(-1\right)}^{j}\gamma_{j}{\hat{x}}_{i1}\left(k-j|k-j\right). \end{array}$$

Then, it is easy to obtain that (19) and (20).

From Ref. [11], it follows that

(27) $$\begin{array}{c} {\hat{y}}_{i1}\left(k|k-1\right)=\hat{Ε}\left[y_{i1}\left(k\right)|Y_{i}^{\left(k-1\right)}\right]=\hat{Ε}{\overset{¯}{C}}_{i}x_{1}\left(k\right)+\eta_{i}\left(k\right)|Y_{i}^{\left(k-1\right)}={\overset{¯}{C}}_{i}{\hat{x}}_{1}\left(k|k-i\right). \end{array}$$

Furthermore, applying (2) yields

(28) $$\begin{array}{c} {\hat{x}}_{i1}\left(k|k\right)=\hat{Ε}\left[x_{1}\left(k\right)|Y_{i}^{\left(k\right)}\right]=\hat{Ε}\left[x_{1}\left(k\right)|Y_{i}^{\left(k-1\right)}\right]+\hat{Ε}\left[{\tilde{x}}_{i1}\left(k|k-1\right){\tilde{y}}_{i1}^{Τ}\left(k|k-1\right)\right]\times {\left\{\hat{Ε}\left[{\tilde{y}}_{i1}\left(k|k-1\right){\tilde{y}}_{i1}^{Τ}\left(k|k-1\right)\right.\right\}}^{-1}{\tilde{y}}_{i1}\left(k|k-1\right)\\ \hat{Ε}\left[{\tilde{x}}_{i1}\left(k|k-1\right){\tilde{y}}_{i1}^{Τ}\left(k|k-1\right)\right]=\hat{Ε}\left[{\tilde{x}}_{i1}\left(k|k-1\right){\left(y_{i1}\left(k\right)-{\hat{y}}_{i1}\left(k|k-1\right)\right)}^{Τ}\right]=P_{i1}\left(k|k-1\right){\overset{¯}{C}}_{i}^{T}\\ \hat{Ε}\left[{\tilde{y}}_{i1}\left(k|k-1\right){\tilde{y}}_{i1}^{Τ}\left(k|k-1\right)\right]=\hat{Ε}\left[\left(y_{i1}\left(k\right)-{\tilde{y}}_{i1}\left(k|k-1\right)\right){\left(y_{i1}\left(k\right)-{\hat{y}}_{i1}\left(k|k-1\right)\right)}^{Τ}\right]\\ ={\overset{¯}{C}}_{i}P_{1}\left(k|k-1\right){\overset{¯}{C}}_{i}^{T}+Q_{\eta i}. \end{array}$$

with ${\tilde{x}}_{i1}\left(k|k-1\right)=x_{1}\left(k\right)-{\hat{x}}_{i1}\left(k|k-1\right)$, ${\tilde{y}}_{i1}\left(k|k-1\right)=$, $y_{1}\left(k\right)-{\hat{y}}_{i1}\left(k|k-1\right)$, *P*_*i*1_(*k|k* − 1)=$Ε\left[\left(x_{1}\left(k\right)-{\hat{x}}_{i1}\left(k|k-1\right)\right.{\left(x_{1}\left(k\right)-{\hat{x}}_{i1}\left(k|k-1\right)\right)}^{Τ}\right]$.

Furthermore, it is obtained that

(29) $$\begin{array}{c} {\hat{x}}_{i1}\left(k|k\right)={\hat{x}}_{i1}\left(k|k-1\right)+P_{i1}\left(k|k-1\right){\overset{¯}{C}}_{i}^{T}\left({\overset{¯}{C}}_{i}P_{i1}\left(k|k-1\right){\overset{¯}{C}}_{i}^{T}+Q_{\eta i}\right).\left[y_{i1}\left(k\right)-{\overset{¯}{C}}_{i}{\hat{x}}_{i1}\left(k|k-1\right)\right]. \end{array}$$

Taking $K_{i1}\left(k\right)=P_{i1}\left(k|k-1\right){\overset{¯}{C}}_{i}^{T}\left({\overset{¯}{C}}_{i}P_{i1}\left(k|k-1\right){\overset{¯}{C}}_{i}^{T}+Q_{\eta i}\right)$as the gain matrix of fractional Kalman filter, we have (19) and (24).

(30) $$\begin{array}{c} x_{1}\left(k\right)-{\hat{x}}_{i1}\left(k|k-1\right)={\tilde{A}}_{\alpha i}x_{1}\left(k-1\right)+J_{i}y_{i1}\left(k-1\right)+{\tilde{w}}_{i1}\left(k-1\right)-\sum_{j=1}^{k}{\left(-1\right)}^{j}\gamma_{j}x_{1}\left(k-j\right)-{\tilde{A}}_{\alpha i}{\hat{x}}_{i1}\left(k-1|k-1\right)+J_{i}y_{i1}\left(k-1\right)+\sum_{j=1}^{k}{\left(-1\right)}^{j}\gamma_{j}{\hat{x}}_{i1}\left(k-j|k-j\right)\\ =\left({\tilde{A}}_{\alpha }-\gamma_{1}\right)\left(x_{1}\left(k-1\right)-{\hat{x}}_{i1}\left(k-1|k-1\right)\right)-\sum_{j=2}^{k}{\left(-1\right)}^{j}\gamma_{j}\left(x_{1}\left(k-j\right)-{\hat{x}}_{i1}\left(k-j|k-j\right)\right)+{\tilde{w}}_{i1}\left(k-1\right). \end{array}$$

With $Ε\left[\left(x_{1}\left(m\right)-{\hat{x}}_{i1}\left(\min-1|\min-1\right)\right.\times {\left(x_{1}\left(m\right)-{\hat{x}}_{i1}\left(\min-1|\min-1\right)\right)}^{Τ}\right]=0$, *m* ≠ *n*, Ε(*x*_1_(*m*)−$\left.Ε\left[\left(x_{1}\left(m\right)-{\hat{x}}_{i1}\left(\min-1|\min-1\right)\right.\times {\left(x_{1}\left(m\right)-{\hat{x}}_{i1}\left(\min-1|\min-1\right)\right)}^{Τ}\right]=0{\tilde{w}}_{i1}^{Τ}\left(k-1\right)\right]=0$, *m*=1, ⋯, *k* − 1.

Then, we have the following equation:

(31) $$\begin{array}{c} P_{i1}\left(k|k-1\right)=Ε\left[\left(x_{1}\left(k\right)-{\hat{x}}_{i1}\left(k|k-1\right)\right){\left(x_{1}\left(k\right)-{\hat{x}}_{i1}\left(k|k-1\right)\right)}^{Τ}\right],\\ =\left({\tilde{A}}_{\alpha i}-\gamma_{1}\right)P_{i1}\left(k|k\right){\left({\tilde{A}}_{\alpha i}-\gamma_{1}\right)}^{Τ}-\sum_{j=2}^{k}\gamma_{j}P_{i1}\left(k-j|k-j\right)\gamma_{j}^{Τ}+Q_{\eta i}. \end{array}$$

so we have (22).

(32) $$\begin{array}{c} x_{1}\left(k\right)-{\hat{x}}_{i1}\left(k|k\right)=x_{1}\left(k\right)-{\hat{x}}_{i1}\left(k|k-1\right)-K_{i1}\left(k\right)\left(y_{i1}\left(k\right)-{\overset{¯}{C}}_{i}{\hat{x}}_{i}\left(k|k-1\right)\right),\\ =x_{1}\left(k\right)-{\hat{x}}_{i1}\left(k|k-1\right)-K_{i1}\left(k\right)\left(x_{1}\left(k\right)+\eta_{i}\left(k\right)-{\overset{¯}{C}}_{i}{\hat{x}}_{i1}\left(k|k-1\right)\right),\\ P_{i1}\left(k|k\right)=E\left(x_{1}\left(k\right)-{\hat{x}}_{i1}\left(k|k\right)\right){\left(x_{1}\left(k\right)-{\hat{x}}_{i1}\left(k|k\right)\right)}^{T},\\ =\left(I-K_{i1}\left(k\right){\overset{¯}{C}}_{i}\right)P_{i1}\left(k|k-1\right){\left(I-K_{i1}\left(k\right){\overset{¯}{C}}_{i}\right)}^{T}+K_{i1}\left(k\right)P_{i1}\left(k|k\right)K_{i1}\left(k\right)=\left(I-K_{i1}\left(k\right){\overset{¯}{C}}_{i}\right)P_{i1}\left(k|k-1\right). \end{array}$$

Then, we have (23). The proof is completed.

**Theorem 2 .**

For the subsystems 2 (2), (10), and (15) with uncorrelated noise noises, we have the local recursive fractional Kalman filter.

(33) $$\begin{array}{c} {\hat{x}}_{2}\left(k|k\right)=T_{3}^{-1}\left(S_{2}S_{1}^{-1}T_{1}-T_{2}\right){\hat{x}}_{1}\left(k|k\right),\\ P_{2}\left(k|k\right)={\left.T_{3}^{-1}\left(S_{2}S_{1}^{-1}T_{1}-T_{2}\right)P_{1}\left(k|k\right)\left[T_{3}^{-1}\left(S_{2}S_{1}^{-1}T_{1}-T_{2}\right)\right]\right)}^{T}. \end{array}$$

**Proof**

Applying the projection theorem, is easy to obtain based on (11).

Using the track fusion algorithm, Theorem 1 and 2, we can easily obtain the two sensor track fusion state estimator.

(34) $$\begin{array}{c} {\hat{x}}_{om}\left(k|k\right)=\left(P_{im}^{-1}\left(k|k\right)+P_{jm}^{-1}\right){\left(k|k\right)}^{-1}\left[P_{im}^{-1}\left(k|k\right){\hat{x}}_{im}\left(k|k\right)+P_{jm}^{-1}\left(k|k\right){\hat{x}}_{jm}\left(k|k\right)\right],m=1,2. \end{array}$$

and the estimation error covariance is

(35) $$\begin{array}{c} P_{0m}\left(k|k\right)={\left(P_{im}^{-1}\left(k|k\right)+P_{jm}^{-1}\left(k|k\right)\right)}^{-1},\;m=1,2. \end{array}$$

If the fusion system is composed of *L* sensors, it can be easily extended to the general form.

**Remark 1 .**

From (39), we have *P*_0*m*_^−1^(*k|k*) > *P*_*im*_^−1^(*k|k*), then we can easily obtain the relationship *P*_*om*_(*k|k*) < *P*_*im*_(*k|k*). It shows the estimation accuracy of track fuser is higher than that of local estimators.

**Theorem 3 .**

For the fractional subsystem 1 (2), (10), and (15) with white uncorrelated noises, we have the track fusion recursive fractional Kalman filter.

(36) $$\begin{array}{c} {\hat{x}}_{01}\left(k|k\right)=P_{01}\left(|kk\right)\sum_{i=1}^{L}P_{i1}^{-1}\left(|kk\right){\hat{x}}_{i1}\left(|kk\right),\\ P_{01}\left(k|k\right)={\left[\sum_{i=1}^{n}P_{i1}^{-1}\left(|kk\right)\right]}^{-1}. \end{array}$$

**Proof**

It is easily obtained by applying the track fusion algorithm to Theorem 1.

**Theorem 4 .**

For the subsystem 2 (2), (10), and (15) with uncorrelated noise noises, we have the track fusion recursive fractional Kalman filter.

(37) $$\begin{array}{c} {\hat{x}}_{02}\left(k|k\right)=T_{3}^{-1}\left(S_{2}S_{1}^{-1}T_{1}-T_{2}\right){\hat{x}}_{01}\left(k|k\right),\\ P_{02}\left(k|k\right)=T_{3}^{-1}\left(S_{2}S_{1}^{-1}T_{1}-T_{2}\right)P_{01}\left(k|k\right){\left[T_{3}^{-1}\left(S_{2}S_{1}^{-1}T_{1}-T_{2}\right)\right]}^{T} \end{array}$$

**Proof**

It is easily obtained based on Theorem 2 and 3.

## 4. Simulation Example Analysis

**Figure 1:**
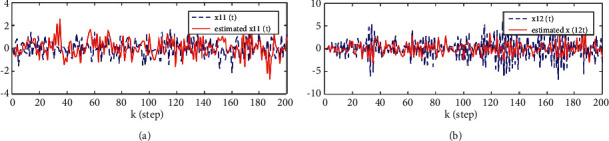
Comparison between the true and estimated values of state for the subsystem 1 based on sensor 1. (a)*x*_1_(*k*) and ${\hat{x}}_{11}\left(k|k\right)$. (b)*x*_2_(*k*) and ${\hat{x}}_{12}\left(k|k\right)$.

**Figure 2:**
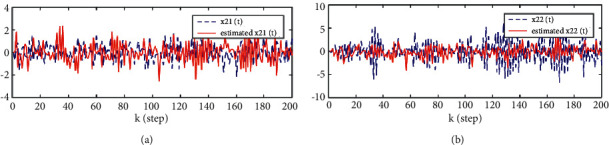
Comparison between the true and estimated values of state for the subsystem 1 based on sensor 2. (a)*x*_1_(*k*) and ${\hat{x}}_{21}\left(k|k\right)$. (b)*x*_2_(*k*) and ${\hat{x}}_{22}\left(k|k\right)$.

**Figure 3:**
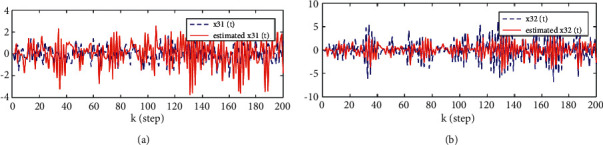
Comparison between the true and estimated values of state for the subsystem 1 based on sensor 3. (a)*x*_1_(*k*) and ${\hat{x}}_{31}\left(k|k\right)$. (b)*x*_2_(*k*) and ${\hat{x}}_{32}\left(k|k\right)$.

**Figure 4:**
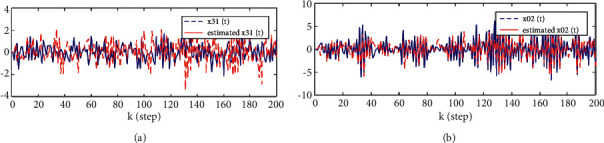
Comparison between the true and track fusion estimated values of state for the subsystem 1. (a)*x*_1_(*k*) and ${\hat{x}}_{01}\left(k|k\right)$. (b)*x*_2_(*k*) and ${\hat{x}}_{02}\left(k|k\right)$.

**Figure 5:**
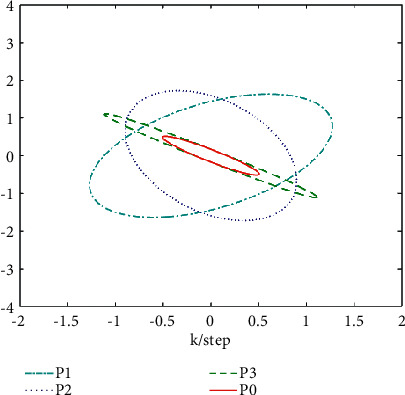
The accuracy comparison of local and track fusion fractional Kalman filter for the subsystem 1 based on covariance ellipse.

**Figure 6:**
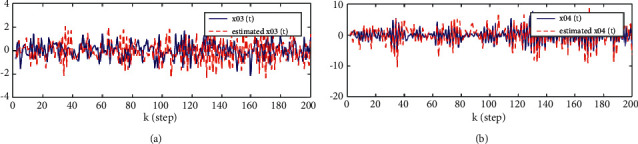
Comparison between the true and track fusion estimated values of state for the subsystem 2. (a)*x*_3_(*k*) and ${\hat{x}}_{03}\left(k|k\right)$. (b)*x*_4_(*k*) and ${\hat{x}}_{04}\left(k|k\right)$.

Considering the canonicality of generalized fractional order systems,

(38) $$\begin{array}{c} \left[\begin{array}{cccc} -2 & 0 & 0 & 0\\ -1 & 1.2 & 0 & 0\\ 1 & 0 & 0 & 0\\ 0.5 & -0.9 & 0 & 0 \end{array}\right]\Delta^{\gamma }x\left(k+1\right)=\left[\begin{array}{cccc} -1 & 0 & 0 & 0\\ -0.38 & 1.08 & 0 & 0\\ 1 & 0.5 & -2 & 0\\ 0 & -1 & -0.7 & 1 \end{array}\right]x\left(k\right)+\left[\begin{array}{c} 0.5\\ 1\\ 0.5\\ 1 \end{array}\right]+w\left(k\right),\\ y_{i}\left(k+1\right)=\left[\begin{array}{cccc} 1 & -2 & 2 & 1 \end{array}\right]x\left(k+1\right)+v_{i}\left(k+1\right),i=1,2,3. \end{array}$$

where *w*(*k*) and *v*(*k*) are uncorrelated white noises with zero means and variances $\left[\begin{array}{cccc} 1 & 0 & 0 & 0\\ 0 & 1 & 0 & 0\\ 0 & 0 & 2 & 0\\ 0 & 0 & 0 & 2 \end{array}\right]$ and *R*_1_ = 0.5, *R*_2_ = 0.5, *R*_3_ = 3, *n*_1_ = 0.8, *n*_2_ = 1.1, $S_{1}=\left[\begin{array}{cc} -2 & 0\\ 1 & -1.2 \end{array}\right]$, $S_{2}=\left[\begin{array}{cc} 1 & 0\\ 0.5 & -0.9 \end{array}\right]$, $T_{1}=\left[\begin{array}{cc} 1 & 0\\ -0.38 & 1.08 \end{array}\right]$, $T_{2}=\left[\begin{array}{cc} 1 & 0.5\\ 0 & -1 \end{array}\right]$, $T_{3}=\left[\begin{array}{cc} -2 & 0\\ -0.7 & 1 \end{array}\right]$, $C_{1}=\left[\begin{array}{cc} 1 & -2 \end{array}\right]$, and $C_{2}=\left[\begin{array}{cc} 2 & 1 \end{array}\right]$. The problem is to find the local descriptor fractional Kalman filter for the subsystem 1 ${\hat{x}}_{i}\left(k|k\right)=\left[{\hat{x}}_{i1}\left(k|k\right),{\hat{x}}_{i2}\left(k|k\right)\right.$ and track fusion descriptor fractional Kalman filter ${\hat{x}}_{0}\left(k|k\right)=\left[{\hat{x}}_{01}\left(k|k\right),{\hat{x}}_{02}\left(k|k\right),{\hat{x}}_{03}\left(k|k\right),{\hat{x}}_{04}\left(k|k\right)\right]$. The simulation results are given by Figures 1–6.

From Theorem 1 and 2, we have the local and track fusion descriptor fractional Kalman filter for the subsystem 1, which are given by Figures 1–4. From Figures 1–3 we find that the descriptor fractional Kalman filter can realize the state estimation for the state of subsystem 1. Compared with Figures 1–3, it is shown that the estimation curve in Figure 4 is closer to the true value curve than that in Figures 1–3. It means that the track fusion algorithm improves the estimation accuracy. From [28], we know that the inclusion relation of covariance ellipses and the size relation of error variance matrices are necessary and sufficient conditions for each other. It shows that the estimation error variance matrix is smaller than that of each local estimation error variance matrices by Figure 5. Based on the track fusion estimator of subsystem, the track fusion fractional Kalman filter for the subsystem 2 is given by Figure 6. In a word, the presented descriptor fractional Kalman filtering algorithm are effective and realizable.

## 5. Conclusions

Based on the existing fractional filtering theory and the knowledge of descriptor systems, this paper normalizes the multisensor fractional order descriptor system, carries out fractional filtering, and then presents the track fusion fractional filters, which provides a new form for the filtering of descriptor systems. The introduction of the track fusion algorithm greatly improves the state estimation accuracy for the multisensor descriptor fractional order systems. Compared with [6], the information fusion state estimation problem for the multisensor descriptor fractional systems is solved. The simulation results show the validity and feasibility of the proposed algorithm.

## Data Availability

The data used to support the findings of this study are available from the corresponding author upon request.
